# Combining high-throughput phenotyping and genome-wide association studies to reveal natural genetic variation in rice

**DOI:** 10.1038/ncomms6087

**Published:** 2014-10-08

**Authors:** Wanneng Yang, Zilong Guo, Chenglong Huang, Lingfeng Duan, Guoxing Chen, Ni Jiang, Wei Fang, Hui Feng, Weibo Xie, Xingming Lian, Gongwei Wang, Qingming Luo, Qifa Zhang, Qian Liu, Lizhong Xiong

**Affiliations:** 1 Britton Chance Center for Biomedical Photonics, Wuhan National Laboratory for Optoelectronics, Huazhong University of Science and Technology, Wuhan 430074, China; 2 National Key Laboratory of Crop Genetic Improvement, National Center of Plant Gene Research, Huazhong Agricultural University, Wuhan 430070, China; 3 MoE Key Laboratory for Biomedical Photonics, Department of Biomedical Engineering, Huazhong University of Science and Technology, Wuhan 430074, China; 4 College of Engineering, Huazhong Agricultural University, Wuhan 430070, China; 5 MOA Key Laboratory of Crop Ecophysiology and Farming System in the Middle Reaches of the Yangtze River, Huazhong Agricultural University, Wuhan 430070, China

## Abstract

Even as the study of plant genomics rapidly develops through the use of high-throughput sequencing techniques, traditional plant phenotyping lags far behind. Here we develop a high-throughput rice phenotyping facility (HRPF) to monitor 13 traditional agronomic traits and 2 newly defined traits during the rice growth period. Using genome-wide association studies (GWAS) of the 15 traits, we identify 141 associated loci, 25 of which contain known genes such as the Green Revolution semi-dwarf gene, *SD1*. Based on a performance evaluation of the HRPF and GWAS results, we demonstrate that high-throughput phenotyping has the potential to replace traditional phenotyping techniques and can provide valuable gene identification information. The combination of the multifunctional phenotyping tools HRPF and GWAS provides deep insights into the genetic architecture of important traits.

## Abstract (web summary)

Next-generation sequencing technology has made the generation of huge amounts of genetic data possible, but phenotype characterization remains slow and difficult. Here the authors develop a high-throughput phenotyping facility for rice that is able to accurately identify and characterize traits related to morphology, biomass and yield.

## Graphical abstract

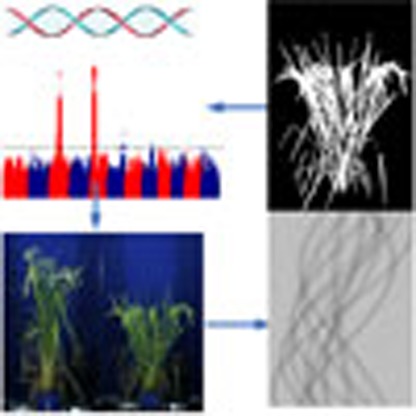

The advent of next-generation sequencing technology has had a major impact on genomics in a short period of time^1^. However, phenomics, a new discipline involving the characterization of the full set of phenotypes of a given species, still lags far behind genomics^2^. Traditional phenotyping tools, which inefficiently measure a limited set of phenotypes, have become a bottleneck in functional genomics and plant breeding studies^3^. The use of multidisciplinary techniques, such as novel imaging sensors, image analysis and robotics, has enabled the development of high-throughput and large-scale noninvasive phenotyping infrastructures^4^,^5^. In previous decades, the key questions of genomics involved why and how to sequence genomes; now, we are facing new challenge: phenomics^2^.

There is a large gap between the present linear increase in global food production and the predicted demand^6^. Rice (*Oryza* sativa landrace) is one of the most important food crops worldwide and has served as a model plant with many advantages, including its abundant natural variation^7^. In recent years, genome-wide association studies (GWAS) using high-throughput sequencing technology have been conducted to dissect the genetic architecture of important traits exhibited by rice^8^,^9^,^10^,^11^. In GWAS, traditional phenotyping is important but laborious, and progress in phenotyping technologies is required to accelerate genetic mapping and gene discovery^12^,^13^.

In the present study, we develop a high-throughput rice phenotyping facility (HRPF) that is able to elucidate traits related to morphology, biomass and yield during the rice growth period and after harvest. Using a combination of HRPF and GWAS, we demonstrate that high-throughput phenotyping has the potential to replace traditional phenotyping and serves as a novel tool for studies of plant genetics, genomics, gene characterization and breeding.

## Results

### Rice automatic phenotyping and yield traits scorer

**Figure 1:**
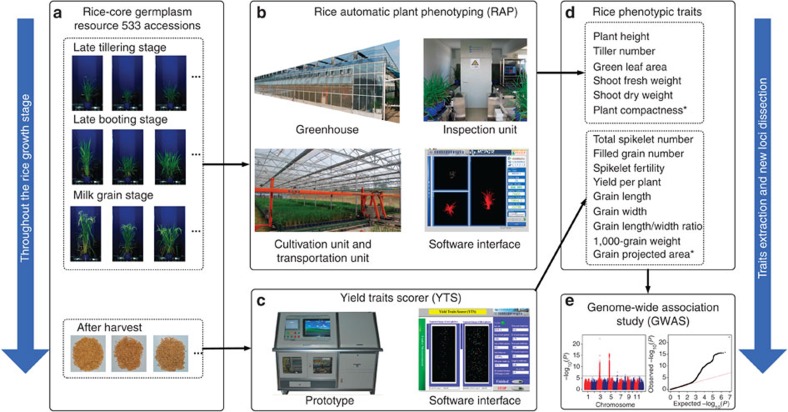
Combination of the HRPF (RAP and YTS) and genome-wide association study (GWAS). To automatically screen the rice-core germplasm resource throughout the growth period (**a**), the entire HRPF was designed with two main elements: a rice automatic plant phenotyping device (RAP, **b**) and a YTS (**c**). These novel phenotyping tools were able to extract not only the traditional agronomic traits but also several novel phenotypic traits (such as plant compactness and grain-projected area). After the rice phenotypic traits (**d**) were extracted with the RAP and YTS, new loci were dissected using GWAS (**e**). *New traits are those that cannot be defined and extracted using traditional measurement techniques.

To enable high-throughput and automatic phenotypic screening of rice germplasm resources and populations throughout the growth period and after harvest (Fig. 1a), a phenotyping facility was designed with two main sections: a rice automatic phenotyping platform (RAP; Fig. 1b) and a yield traits scorer (YTS; Fig. 1c). The RAP, which included greenhouse, transportation and inspection units, was a highly integrated facility that could achieve high-throughput screening of rice plants. The inspection unit of the RAP included two devices: a colour-imaging device and a linear X-ray computed tomography (CT). The colour imaging (also called optical imaging) was designed to non-destructively extract morphology-related traits (plant height, green leaf area and plant compactness; Fig. 1d) and biomass-related traits (shoot fresh weight and shoot dry weight; Fig. 1d). After colour image acquisition and two-dimensional (2D) image processing, 32 features, including plant height, plant compactness and other morphological and texture features, were extracted for each plant. The features were then combined with the manual measurements of shoot fresh weight, shoot dry weight and green leaf area of the same rice accessions to generate the best model for predicting these three traits using feature grouping and all-subset regression. The linear X-ray CT was used to automatically measure the tiller number as described in our previous study^14^.

After harvest, the rice yield-related traits (total spikelet number, filled grain number, spikelet fertility, yield per plant, 1,000-grain weight and grain shape and size; Fig. 1d) are often measured by researchers. In this study, we developed an engineering prototype of the YTS to automatically extract these traits. The detailed operating procedures of the RAP and YTS are provided in the Methods section and Supplementary Videos 1 and 2.

### Performance evaluation of the RAP and YTS

**Figure 2:**
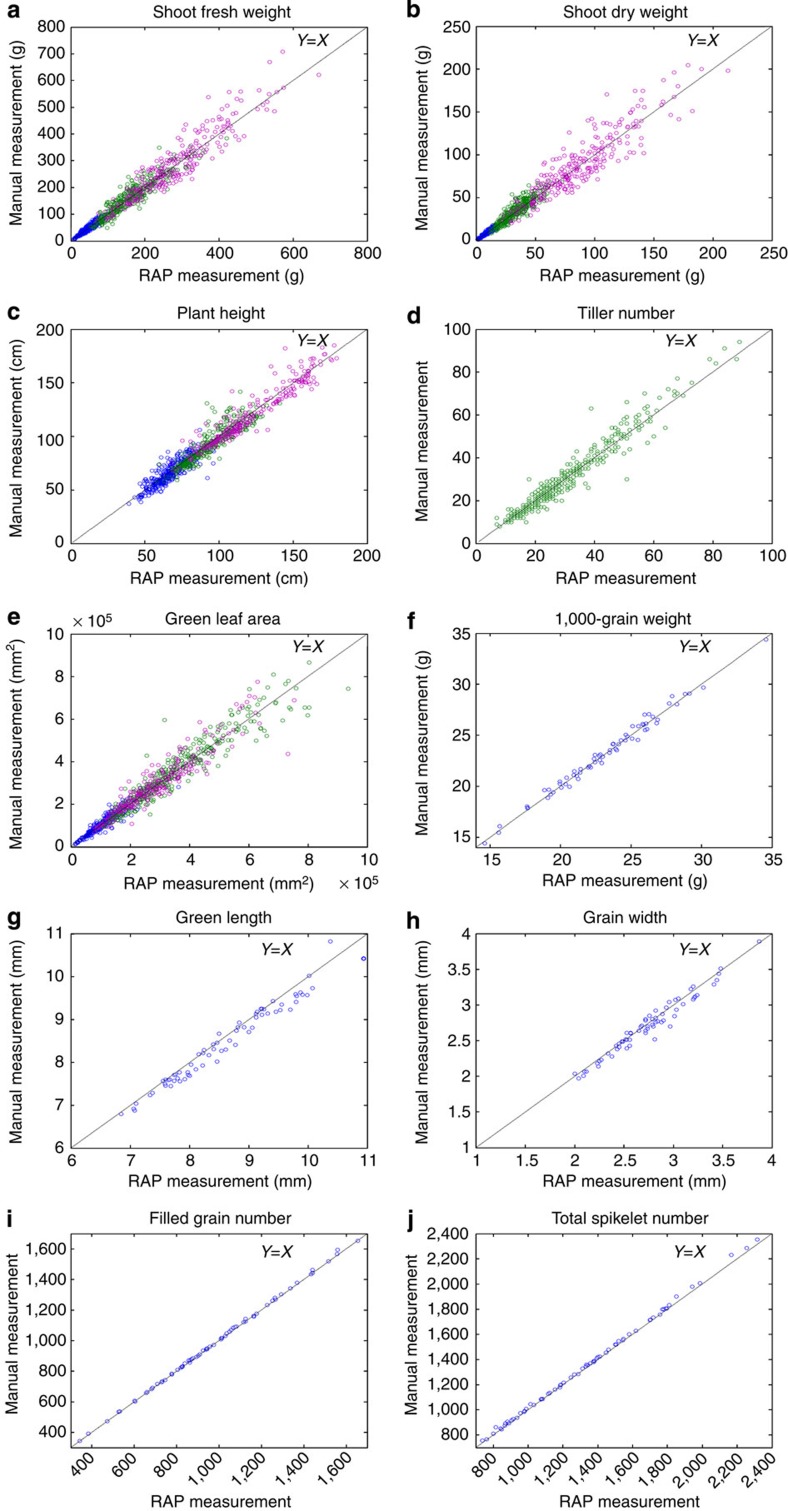
Scatter plots of 10 RAP/YTS measurements versus manual measurements. (**a**) Shoot fresh weight, (**b**) shoot dry weight, (**c**) plant height, (**d**) tiller number and (**e**) green leaf area; the blue plots, green plots and purple plots represent the measurements at the late tillering stage, late booting stage and milk grain stage, respectively. Other scatter plots indicate the YTS measurements versus manual measurements for (**f**) 1,000-grain weight, (**g**) grain length, (**h**) grain width, (**i**) filled grain number and (**j**) total spikelet number. The details of the 10 rice phenotypic trait measurement accuracies are shown in Supplementary Table 1.

The overall evaluation experiment using the RAP and YTS is described in Supplementary Fig. 1a. During three critical growth and development stages (late tillering stage, late booting stage and milk grain stage), five phenotypic traits were measured by the RAP and manual methods. Scatter plots showing manual versus automatic measurements of the traits are shown in Fig. 2. For all the testing sets, the *R*^2^ and mean absolute percentage error of the five traits ranged from 0.82 to 0.90 and 5.59 to 13.28%, respectively. As shown in Supplementary Fig. 1b, when continuously operated (24 h per day), the total throughput of the RAP was 1,920 pot-grown rice plants out of a total greenhouse capacity of 5,472 pots (Supplementary Fig. 1c).

To extract the green leaf area, shoot fresh weight and shoot dry weight, half of the rice samples were randomly selected as a training set for model construction, and the prediction performance of the model was evaluated using the testing set and cross-validation. To select effective predictors for these three traits, all possible regressions were performed using Akaike’s information criterion, the adjusted coefficient of determination (adjusted *R*^*2*^) and the prediction error sum of squares (PRESS statistic)^15^,^16^. Four models, including Model A (using area as the indicator, which is an easily extracted feature), Model AM (using area and one morphological feature as indicators), Model AT (using area and one texture feature as indicators) and Model ATM (using area, one morphological feature, and one texture feature as indicators), were selected and compared. The best model was required to perform noticeably better than those using fewer predictors. The model determination details are shown in Supplementary Fig. 2 and Supplementary Fig. 3. Supplementary Table 1 shows the selected models and their measurement accuracies.

After harvest, 514 accessions (four replicates of each accession) from rice-core germplasm resources were evaluated with the YTS, and 68 accessions were randomly selected and measured manually to estimate the measurement accuracy of the YTS. The *R*^2^ and mean absolute percentage error of the yield traits were 0.96–0.99 and 0.89–2.52%, respectively. The measurement accuracies of the YTS are listed in Supplementary Table 1. Considering the time required to feed spikelets and to retrieve the filled spikelets, the efficiency of the YTS is ~1 min per plant.

### GWAS with the RAP and YTS

**Figure 3:**
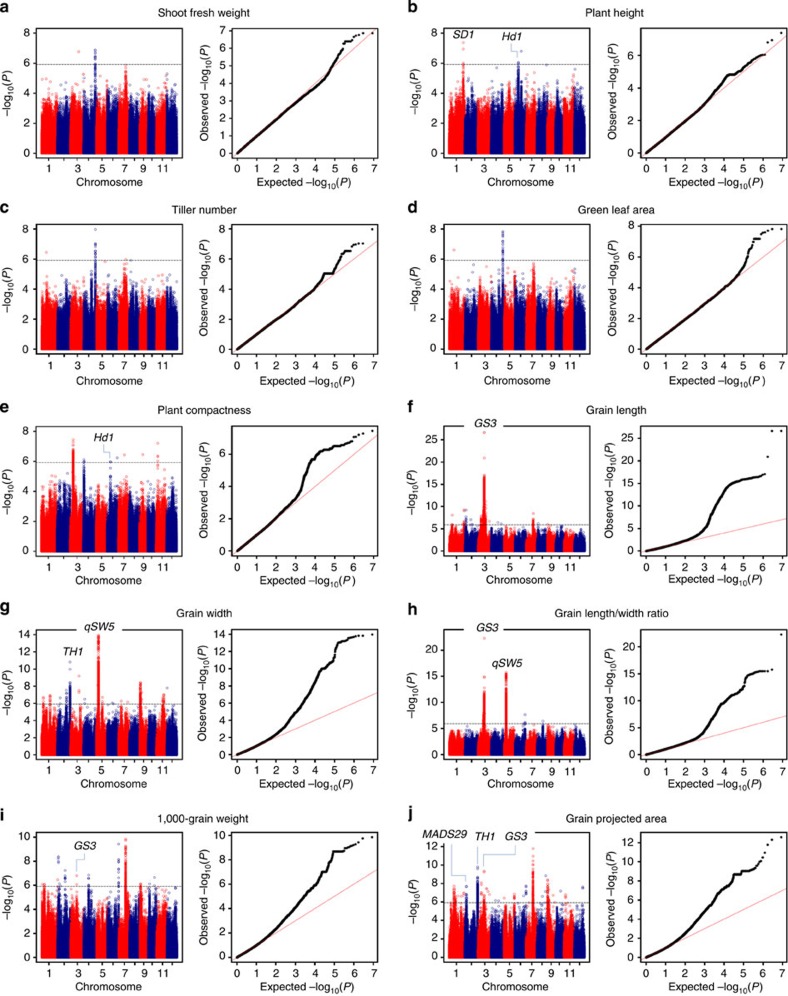
Genome-wide association studies of five traits at the late booting stage measured by the RAP and five yield-related traits measured by the YTS. Manhattan plots (left) and quantile-quantile plots (right) for shoot fresh weight (**a**), plant height (**b**), tiller number (**c**), green leaf area (**d**) and plant compactness (**e**) measured by the RAP, and grain length (**f**), grain width (**g**), grain length/width ratio (**h**), 1,000-grain weight (**i**) and grain-projected area (**j**) measured by the YTS. The sample sizes are 402 for the five traits measured by RAP (**a**–**e**), and the sample sizes are 514 for five yield traits measured by YTS (**f**–**j**). The *P* values are computed from a likelihood ratio test with a mixed-model approach using the factored spectrally transformed linear mixed models (FaST-LMM) programme. For Manhattan plots, −log_10_ *P* values from a genome-wide scan are plotted against the position of the SNPs on each of 12 chromosomes, and the horizontal grey dashed line indicates the genome-wide suggestive threshold (*P*=1.21 × 10^−6^). For quantile-quantile plots, the horizontal axis shows −log_10_-transformed expected *P* values, and the vertical axis indicates −log_10_-transformed observed *P* values. The names of known related genes are shown above the corresponding association peaks.

After establishment of the phenotyping platform, we performed GWAS across 529 diverse *O. sativa* accessions for 15 traits. In contrast to previous related studies, these traits were measured automatically by the RAP and YTS instead of performing manual measurements^8^,^9^. Using a Bonferroni correction based on the effective numbers of independent markers^17^, the *P* value thresholds were 1.21E–06 and 6.03E–08 (suggestive and significant, respectively) for the entire population^18^. In our study, only the associations that exceeded the *P* value thresholds with clear peak-like signals were considered. With the significance threshold set, we identified 57 loci, including 15 loci associated with four traits measured by the RAP and 42 loci with five traits measured by the YTS (Supplementary Data 1). According to the suggestive threshold, 138 associated loci were identified; of these, 49 were associated with six traits measured by the RAP and 89 were associated with five yield-related traits (Supplementary Data 1). Manhattan plots and quantile-quantile plots for the 15 traits at different stages are shown in Fig. 3 and Supplementary Figs 4–8.

Certain loci were simultaneously detected for different traits. For example, a lead single nucleotide polymorphism (SNP) located at bp 2,578,017 on chromosome 1 was associated with shoot fresh weight, shoot dry weight and green leaf area at the late tillering stage. Lead SNPs at 22 associated loci passing the suggestive threshold for seven traits (plant height, plant compactness, grain length, grain width, grain length/width ratio, 1,000-grain weight and grain-projected area) and lead SNPs at another 3 loci with clear peak-like association signals that failed to pass but were close to the suggestive threshold were linked to known related genes (Supplementary Note 3). Among these SNPs, three associated with plant height were linked to *SD1*^19^,^20^,^21^ (the Green Revolution semi-dwarf gene), *Hd1*^22^ and *OsGH3-2*^23^, which were previously reported to affect plant height; one associated with plant compactness, a new morphological trait, was linked to *Hd1*^21^. For yield-related traits, lead SNPs at 21 associated loci were close to *GS3*^24^,^25^, *qSW5*^26^, *TH1*^27^, *MADS29*^28^, *DST*^29^ and *OsPPKL3*^30^, genes that are known to regulate grain size or yield in rice. In addition, a large number of associated loci had not been previously reported (Supplementary Note 4; Supplementary Tables 13 and 14).

### Comparison of GWAS results from three phenotyping methods

**Figure 4:**
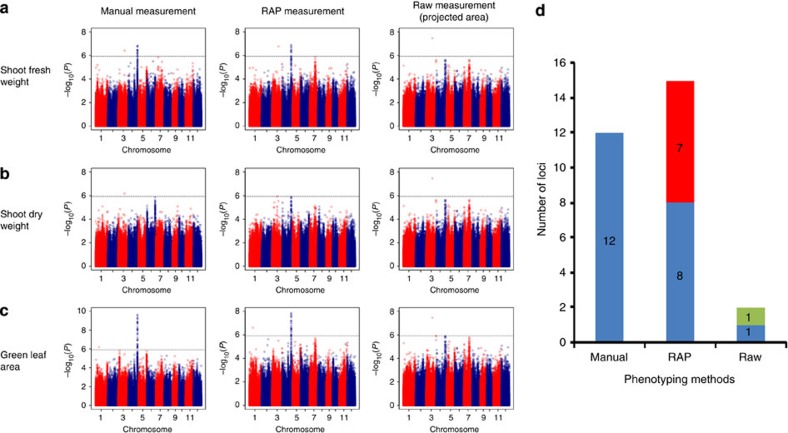
Comparison among GWAS results using three phenotyping methods for shoot fresh weight, shoot dry weight and green leaf area. The three phenotyping methods included manual measurement, RAP measurement and raw measurement. The RAP measurement is the predicted value calculated by the raw features and the selected models (shown in Supplementary Fig. 3). The raw measurement is the projected area calculated by the number of foreground pixels, which is easily extracted without modelling. Manhattan plots for shoot fresh weight (**a**), shoot dry weight (**b**) and green leaf area (**c**) using manual measurement (left), RAP measurement (middle) and raw measurement (right; the projected area was calculated by the number of foreground pixels) at the late booting stage. (**d**) Blue bars indicate associated loci detected by manual measurement. Red bars and green bars indicate specific loci detected by RAP measurement and raw measurement, respectively. The sample sizes of all the three traits are 402. The *P* values are computed from a likelihood ratio test with a mixed-model approach using the factored spectrally transformed linear mixed models (FaST-LMM) programme. For Manhattan plots, −log_10_ *P* values from a genome-wide scan are plotted against the position of the SNPs on each of 12 chromosomes, and the horizontal grey dashed line indicates the genome-wide suggestive threshold (*P*=1.21 × 10^−6^).

In the RAP measurements, after the raw features were extracted, optimized models were chosen to infer shoot fresh weight, shoot dry weight and green leaf area (Supplementary Fig. 3). To evaluate the performance of the RAP with regard to loci identification for the three traits, we compared the RAP measurement, the manual measurement and the raw measurement. The raw measurement is the projected area calculated by the number of foreground pixels, which is easily extracted without modelling. We conducted GWAS for the three traits using these different measurement methods (Fig. 4d; Supplementary Table 15). With the suggestive *P* value thresholds adopted, 12 and 15 associated loci were detected by manual and RAP measurements, respectively. For the raw measurements, however, only two associated loci were detected. For the three traits, 8 of 12 loci detected by manual measurement were also detected by the RAP, whereas only one locus was detected by the raw measurement. We used the GWAS results for the three traits at the late booting stage as an illustration to provide a detailed comparison (Fig. 4). On the basis of Manhattan plots, the GWAS results of the three traits measured by the RAP were consistent with those obtained by manual measurement, whereas the raw measurements of shoot fresh weight and green leaf area failed to detect any associated loci. As shown in Supplementary Table 15, among the three associated loci detected by manual measurement, two were also detected by the RAP, whereas no loci were detected by raw measurement. Detailed information comparing Manhattan and quantile-quantile plots of the four traits at other stages is provided in Supplementary Figs 4–8.

### Comparison of rice accessions for two new traits

**Figure 5:**
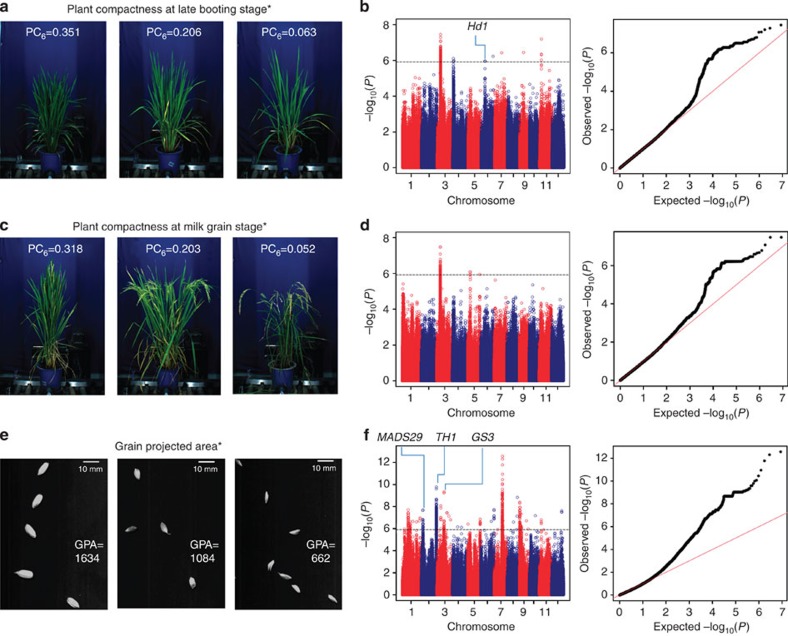
Comparison of rice accessions exhibiting different plant compactness values and grain-projected areas. Representative rice accessions exhibiting different plant compactness values at late booting stage (**a**), different plant compactness values at milk grain stage (**c**), and the grain-projected area (**e**). Manhattan plots (left) and quantile-quantile plots (right) for plant compactness at late booting stage (sample size=402) (**b**), plant compactness at milk grain stage (sample size=269) (**d**), and grain-projected area (sample size=514) (**f**). The *P* values are computed from a likelihood ratio test with a mixed-model approach using the factored spectrally transformed linear mixed models (FaST-LMM) programme (*P*=1.21 × 10^−6^). *New traits are those that cannot be defined and extracted using traditional measurement techniques.

In addition to the traditional agronomic traits, new traits, including plant compactness and grain-projected area, can be extracted by the RAP and the YTS, respectively. Plant compactness reflects plant density and plant architecture, and a more detailed description of plant compactness is provided in the Supplementary Note 2. As shown in Fig. 5a,c, the plants became more compact and the leaves became more upright with increases in plant compactness. Plant compactness provided meaningful information on plant architecture in addition to the commonly recognized traits (such as plant height, tiller number and green leaf area) (Supplementary Fig. 9). This was also the reason that plant compactness was chosen to improve the biomass and leaf area prediction. Seven and four loci were associated with plant compactness at the late booting stage and the milk grain stage, respectively (shown in Fig. 5b,d; Supplementary Data 1). Grain-projected area can be effectively extracted by the YTS and overcomes the limitations inherent to the manual measurement of grain size (Fig. 5e). Traditionally, grain size, which is one of the key component traits for grain yield, is evaluated based on grain length and width. Grain-projected area is a 2D projected image of grain and is a composite trait reflecting both the grain length and width. Several known loci associated with grain size, such as *GS3*^24^,^25^, *MADS29*^28^ and *TH1*^27^, and 24 new loci were detected with grain-projected area (shown in Fig. 5f; Supplementary Data 1).

## Discussion

For biomass (shoot fresh weight and shoot dry weight) prediction, noticeable improvement was achieved by adding morphological features or texture features to the model. Model AM generally performed better than Model AT, with the exception of shoot fresh weight at the late tillering stage. This finding indicated that morphological features were more significant in predicting rice biomass than texture features. The reason that Model AT outperformed Model AM in predicting fresh weight at the late tillering stage may be that the overlap was not significant and the influence of specific organ weight exceeded that of the overlap. After booting, the overlap was more influential. Except for dry weight prediction at the late booting stage, Model ATM showed no noticeable improvement in performance over Model AM. This was because differences in growth status among individual plants during the late booting stage are larger than that during the other three growth periods. Similar conclusions were observed for green leaf area prediction. Noticeable improvement was achieved by adding morphological features or texture features to the model. In addition, Model AM generally performed better than Model AT. The colour of panicles is very similar to that of leaves; thus, the extracted regions of images included both panicles and leaves. To address this problem, a texture feature was added to the model to help reflect the variation and the distribution of the grey level in the image. The addition of the texture feature significantly improved the predictive performance of the model.

From the comparison of the GWAS results with the three different phenotyping methods, we found that the RAP provided a relatively more complete representation than manual measurements in dissecting genetic architecture, and that raw measurement did not have sufficient power to study relatively complex traits such as shoot fresh/dry weight and green leaf area. Compared with the use of only the original features, the optimized model plus the original features will benefit the dissection of the genetic architecture of complex traits. Moreover, eliminating the G × E effects was the first and key step in our phenotyping experiment. All the rice accessions were planted in the greenhouse under the same conditions, and each pot was loaded with equivalent soil and fertilizer, as shown in Supplementary Video 1. Eliminating some outliers in the phenotypic data was another key pre-processing step before GWAS analysis. The improvement after eliminating the outliers is shown in Supplementary Fig. 10.

Although genomics has been advancing very rapidly, traditional plant phenotyping lags far behind current genotyping techniques such as sequencing. To relieve this bottleneck, our work describes a combination of high-throughput phenotyping and GWAS to unlock genetic information coded in the rice genome that controls complex traits and demonstrates the feasibility of replacing laborious manual phenotyping with objective, efficient and non-destructive phenotyping tools such as an HRPF. Our study also demonstrates that for complex traits (such as shoot fresh/dry weight and green leaf area), the RAP better dissected the gene architecture of phenotypic traits than did the raw measurement of these traits. In addition to the traditional traits identified by manual measurement, novel traits (such as plant compactness and grain-projected area, which have obvious implications for planting density and yield) can be specifically phenotyped using the HRPF. With appropriate modifications to image analysis, we anticipate that the combination of the HRPF and GWAS can be used for a wide spectrum of other plant species to determine genetic architecture and provide insights into basic biological processes. As a replacement for traditional phenotyping, the HRPF represents a novel tool that can facilitate major advances in plant functional genomics and crop breeding.

## Methods

### Plant material and experiment design

In our study, 533 *O. sativa* landrace and elite accessions were genotyped (Supplementary Note 5). The basic accession information is shown in Supplementary Data 2. Paired-end 90-bp reads were obtained using the Illumina HiSeq 2000 platform and covered ~1 Gb of the rice genome for each of the 533 accessions after removing adapter contamination and low-quality reads. These sequence reads were aligned to the rice reference genome (the assembly release version 6.1 of genomic pseudomolecules of japonica cv. Nipponbare was downloaded from Michigan State University (http://rice.plantbiology.msu.edu/)) to build the consensus genomic sequence of each accession, and SNP identification was based on the discrepancies between the consensus sequence and the reference genome. Among these accessions, three with severe heterozygosity and one with a low mapping rate (10%) were excluded from the subsequent analysis. For the missing genotype imputation, the linkage disequilibrium-*k*-nearest neighbor (LD-KNN) algorithm was used instead of the KNN algorithm, which has been previously reported^8^. The detailed procedure of genome sequencing, alignment, genotype calling and missing genotype imputation was described in a previous study^31^. The experimental design used to acquire the 15 phenotypic traits for the GWAS and to evaluate the measurement accuracy of the RAP and the YTS throughout the rice growth stages is shown in Supplementary Fig. 1a.

### Operation of the RAP

As shown in Supplementary Video 1 and Supplementary Fig. 1b, when the inspection task starts, the RAP work flowchart includes the following steps: (1) one group (24 pot-grown rice plants; G1) is transported to the industrial conveyor via an automated guided vehicle; (2) the 24 rice plants are transported to the inspection unit; (3) the 24 rice plants are continuously screened with the X-ray CT device and colour-imaging device, while another group (G2) is delivered to the conveyor; and (4) after all of the initial 24 rice plants are inspected, the next group (G2) is transported to the inspection unit, and the first group (G1) is transported back to the greenhouse with the automated guided vehicle. The inspection unit workstation, with control software developed using LabVIEW 8.6 (National Instruments, USA), was designed for image acquisition, image processing, trait storage and communication with a programmable logic controller. The main specifications of the RAP inspection unit are shown in Supplementary Table 12, and additional details of the X-ray CT were reported in our previous work^14^.

### Operation of the YTS

As shown in Supplementary Video 2, the threshed spikelets were placed into the electrovibrating feeder, and the feeder sent the spikelets onto the first conveyor. The monochrome line-array camera captured images of the grains, and the total spikelet number was determined. The spikelet then passed through a wind separator, and the unfilled spikelets were blown away. The filled spikelets were delivered to the second conveyor, and another monochrome line-array camera acquired the images from which the filled spikelet number, spikelet fertility, grain length, grain width, grain length/width ratio and grain-projected area were obtained. After the filled spikelets were collected using the auto-weighing balance, traits including yield per plant and 1,000-grain weight were calculated and recorded. The key components of the YTS are shown in the seed-evaluation accelerator (SEA) inspection unit of our previous work^32^.

### Extraction of phenotypic traits by the RAP and YTS

The specified imaging techniques used in the RAP and YTS are shown in Supplementary Table 17. For each plant, after 12 side-view colour images and 1 X-ray sinogram image were captured and analysed, 33 features, including projected area (*A*), 25 morphological features and 7 texture features, were extracted (Supplementary Fig. 2). As shown in Supplementary Fig. 3, after manual green leaf area or biomass measurements were obtained, several models were built and the best models were chosen for prediction of the green leaf area or biomass. More details about the feature extraction and model selection processes can be found in Supplementary Tables 2–11 and Supplementary Notes 1–2.

### Genome-wide association study

In our association panel containing 529 accessions, a total of 4,358,600 SNPs (minor allele frequency ≥0.05; the number of accessions with minor alleles ≥6) were used in our GWAS for 15 traits. A mixed-model approach was implemented using the factored spectrally transformed linear mixed models (FaST-LMM) programme^33^ with genetic similarities used to estimate random effects. The genetic similarities were defined as the identity genotype proportion of 188,165 evenly distributed random SNPs across the entire rice genome for each pair of individuals^34^. The effective number of independent markers (*N*) was calculated using the GEC software tool^17^ (Supplementary Table 16). Suggestive (1/*N*) and significant (0.05/*N*) *P* value thresholds were set to control the genome-wide type 1 error rate^17^,^18^,^35^. The *P* value thresholds were 1.21E–06 and 6.03E–08 (suggestive and significant, respectively) for the entire population. The LD statistic *r*^2^ based on haplotype frequencies was calculated using Plink^36^. To identify independent lead SNPs of association signals, SNPs passing the *P* value threshold were further clumped to remove the dependent SNPs caused by LD (*r*^2^>0.25) using the clumping function in Plink^36^,^37^.

## Author contributions

W.Y., Z.G., C.H., L.D. and G.C. designed the research, performed the experiments, analysed the data and wrote the manuscript. N.J., W.F. and H.F. also performed the experiments. W.X., X.L. and G.W. provided the rice materials and sequence data. Q.Luo. and Q.Z. initiated the project on the construction of a phenotyping platform. Q.Liu. and L.X. supervised the project, designed the research and wrote the manuscript.

## Additional Information

**How to cite this article:** Yang, W. *et al.* Combining high-throughput phenotyping and genome-wide association studies to reveal natural genetic variation in rice. *Nat. Commun.* 5:5087 doi: 10.1038/ncomms6087 (2014).

## Supplementary Material

Supplementary Figures, Supplementary Tables, Supplementary Notes and Supplementary References Supplementary Figures 1-10, Supplementary Tables 1-17, Supplementary Notes 1-5 and Supplementary References

Supplementary Data 1 Genome-wide association loci exceeding the suggestive P value threshold in our GWAS using RAP/YTS

Supplementary Data 2 The basic information of 533 accessions.

Supplementary Movie 1 The detailed operating procedures of RAP

Supplementary Movie 2 The detailed operating procedures of YTS.
