# Supplementary material for: Combining high-throughput phenotyping and genome-wide association studies to reveal natural genetic variation in rice
Source: Nat Commun. 2014 Oct 8;5:5087. doi: 10.1038/ncomms6087 (PMC4214417; doi:10.1038/ncomms6087)
Supplement: Supplementary Figures, Supplementary Tables, Supplementary Notes and Supplementary References — Supplementary Figures 1-10, Supplementary Tables 1-17, Supplementary Notes 1-5 and Supplementary References [file ncomms6087-s1.pdf]

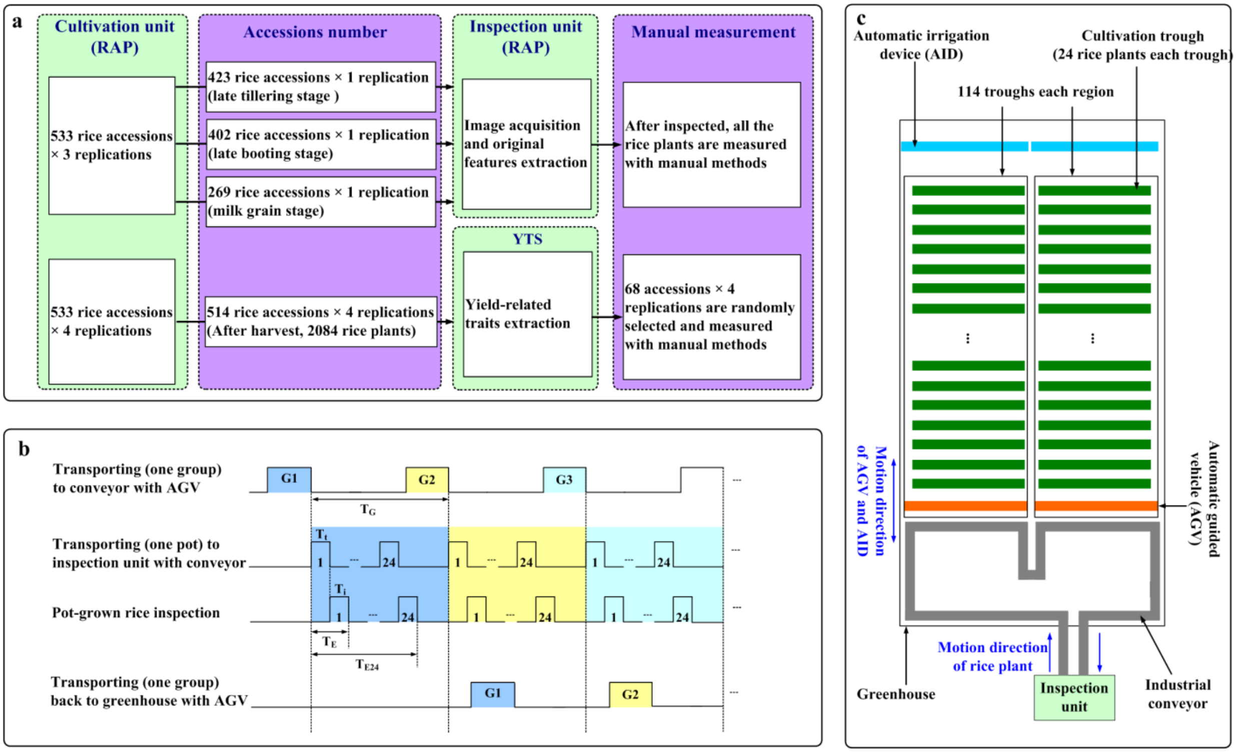

Supplementary Figure 1 Experimental design, workflow chart, and layout of the RAP. a, A total of 3731 rice plants (533 accessions with 7 replicates) were grown in the greenhouse. Due to the growth periods, the 533 accessions were not synchronous, and to ensure that the inspection task at each growth stage was accomplished within approximately one month, not all rice accessions were inspected with RAP. A total of 423, 402, and 269 rice accessions at late tillering stage, late booting stage, and milk grain stage, respectively, were automatically (with RAP) and manually measured. After harvest, 2056 rice plants (514 accessions with 4 replicates) were threshed and then inspected with YTS to extract 9 yield-related traits. To evaluate the measurement accuracy of the YTS, 68 accessions (with 4 replicates) were randomly chosen and manually measured. **b**, As shown in the RAP workflow chart, the time consumption of rice transportation (T_t) and rice inspection (T_i) of each pot was 9 s and 21 s, respectively. Approximately 720 s (denoted by T_{E24}) was required to complete the inspection of one group. The time interval required for the AGV to deliver the group to the conveyor (denoted by T_G, approximately 1080 s) was longer than T_{E24}. Therefore, when continuously

operated 24 hours per day, the total throughput of RAP was 80 groups, equivalent to 1920 pot-grown rice plants per day. **c**, Pot-grown rice plants (24 plants in each row) were placed on one cultivation trough, which was used for AGV lifting or laying down. There were two regions in the greenhouse, and each region contained 114 troughs; thus the capacity of the greenhouse was designed to hold 5472 pots. In addition to the 228 cultivation troughs, 2 AGVs, industrial conveyors with 80 m, and 1 inspection unit, there were two automatic daily irrigation devices. Once the irrigation time and frequency were set, according to the transport and inspection tasks, watering began automatically.

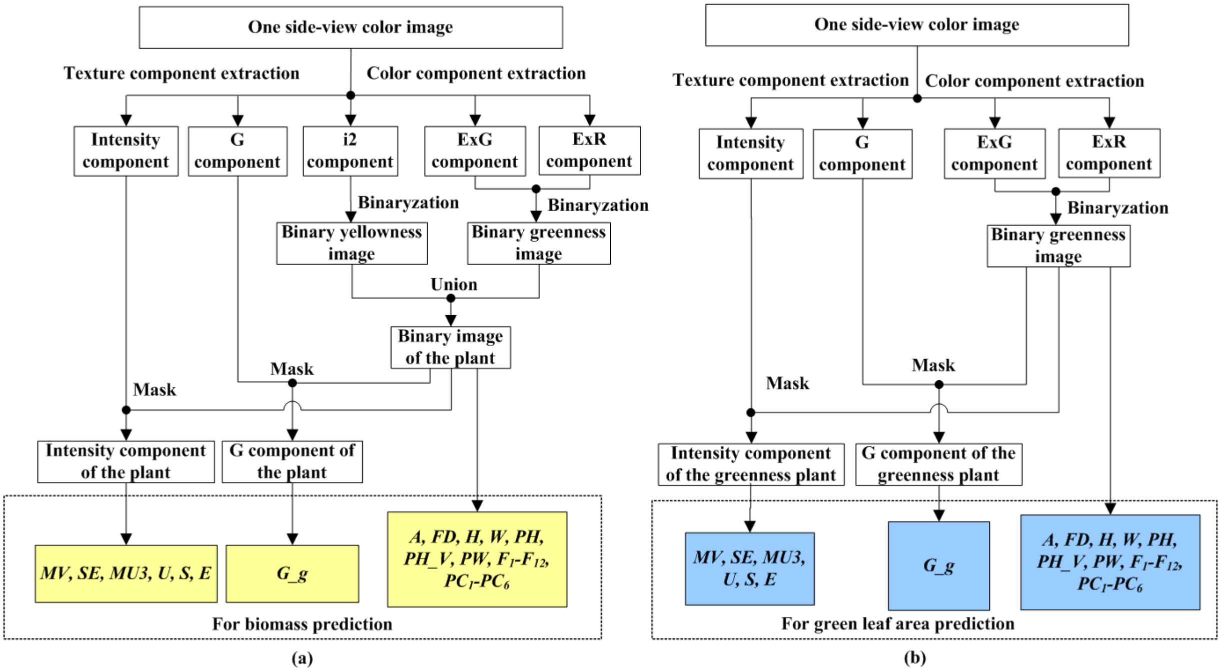

Supplementary Figure 2 Diagram of image processing and feature extraction for one side-view color image. The ExG component, ExR component, and i2 component of the RGB image were extracted; the ExG component and ExR component were used to extract green part of the plant, and the i2 component was used to extract yellow part of the plant (ExG, ExR, and i2 are defined in Supplementary Note 1). A fixed threshold was applied to the ExG image to identify bright objects, and a fixed threshold was applied to the ExR image to identify dark objects. By applying an 'AND' operation to the two binary images, the green plant region was extracted, and the soil in the pot was not identified as plant material. Similarly, a fixed threshold was applied to the i2 component for image binarization. Due to the presence of undesirable spots in the binary image, a processing step was employed to remove small areas and regions with areas smaller than a predefined threshold. The union of the binary greenness image and binary yellowness image were computed to obtain the binary image of the plant, from which the projected area (*A*) and 25 morphological features were extracted. Using the binary plant image as the mask, the G component and intensity component of the plant

were extracted. Similarly, the G component and the intensity component of plant greenness were extracted using the binary ExG image. In the end, G_g was computed from the G component and 6 histogram features were calculated from the intensity component.

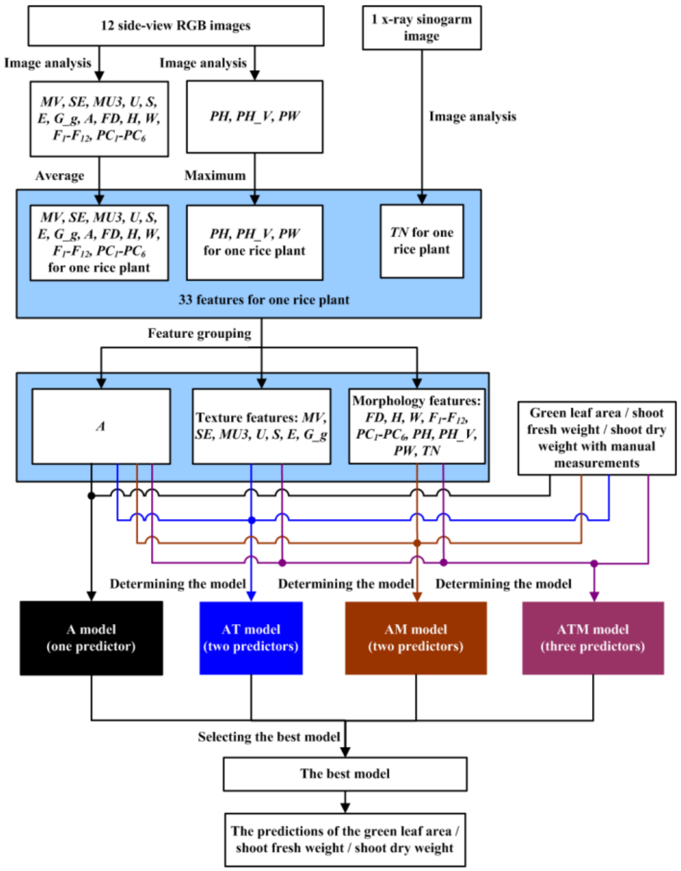

Supplementary Figure 3 Diagram of feature extraction and modeling for green leaf area, shoot fresh weight, and shoot dry weight prediction. One rice plant was horizontally rotated 360 degrees, during which the plant was photographed from 12 angles (one image every 30 degrees). After image analysis, 33 features, including projected area (*A*), 25 morphological features, and 7 texture features, were extracted for each plant. The definitions of the features are provided in Supplementary Table 2. The 33 features were grouped into 4 models. To determine the effective predictors for green leaf area, shoot fresh weight, and shoot dry weight, all possible regressions were performed using Akaike's information criterion (AIC), the adjusted coefficient of determination (adjusted R^2), and the prediction error sum of squares (PRESS statistic). The subset with the lowest PRESS statistic and AIC and the highest adjusted R^2 was selected for further consideration. Four models, including Model A (using area as the indicator, which is an easily extracted feature), Model AM

(using area and 1 morphological feature as indicators), Model AT (using area and 1 texture feature as indicators), and Model ATM (using area, 1 morphological feature, and 1 texture feature as indicators), were selected. The adjusted R^2, PRESS statistic, AIC, coefficient of determination (R^2), root mean squared error (RMSE), and mean absolute percentage error (MAPE) for the training set and testing set and the 5-fold cross-validation were used for further model comparison. Generally, the model with more predictors performed better. However, according to the principle of Occam's Razor, the model with fewer predictors was preferred for its simplicity and generalizability. In this research, the best model should meet the requirement that its performance is noticeably significantly better than that of the models with the fewest predictors.

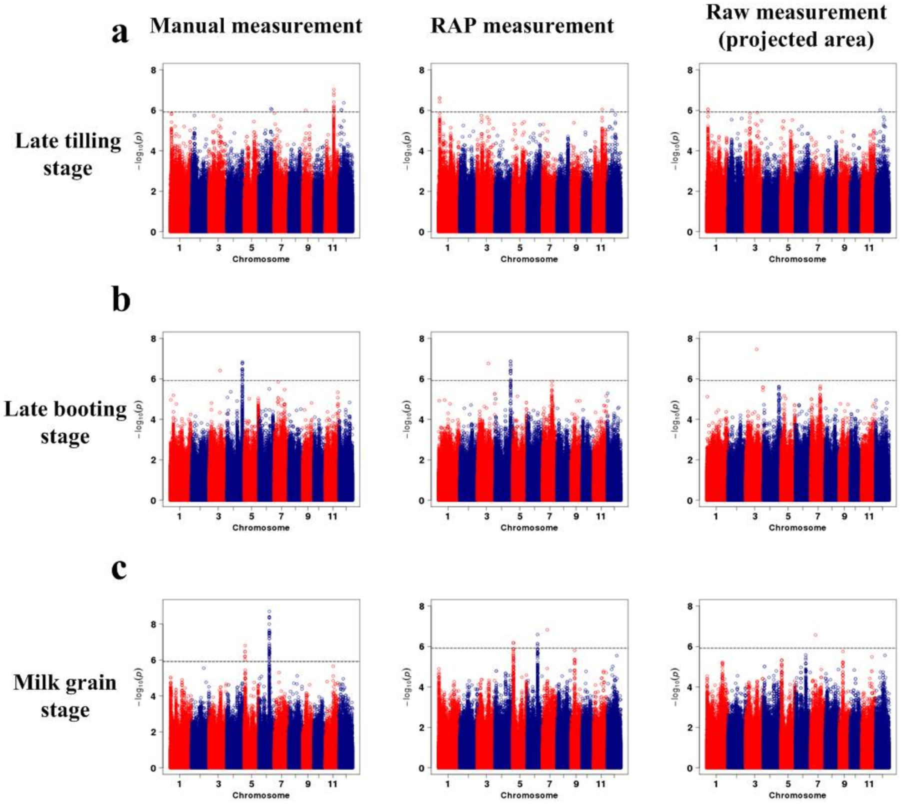

Supplementary Figure 4 Genome-wide association studies of shoot fresh weight at three developmental stages using different phenotyping methods. Manhattan plots for shoot fresh weight at the late tillering stage (a), late booting stage (b), and milk grain stage (c) using the method of manual measurement (left), RAP measurement (middle), and raw measurement (right; the raw feature calculated by the projected area of the entire rice plant, which is easily extracted without modeling). For Manhattan plots, -log10 *P* values from a genome-wide scan were plotted against the position of the SNPs on each of the 12 chromosomes and the horizontal gray dashed line indicates the genome-wide suggestive threshold ($P = 1.21 \times 10^{-6}$). Sample sizes are 423 (a), 402 (b), and 269 (c), respectively. The *P* values are computed from a likelihood ratio test with a mixed-model approach using the factored spectrally transformed linear mixed models (FaST-LMM) program.

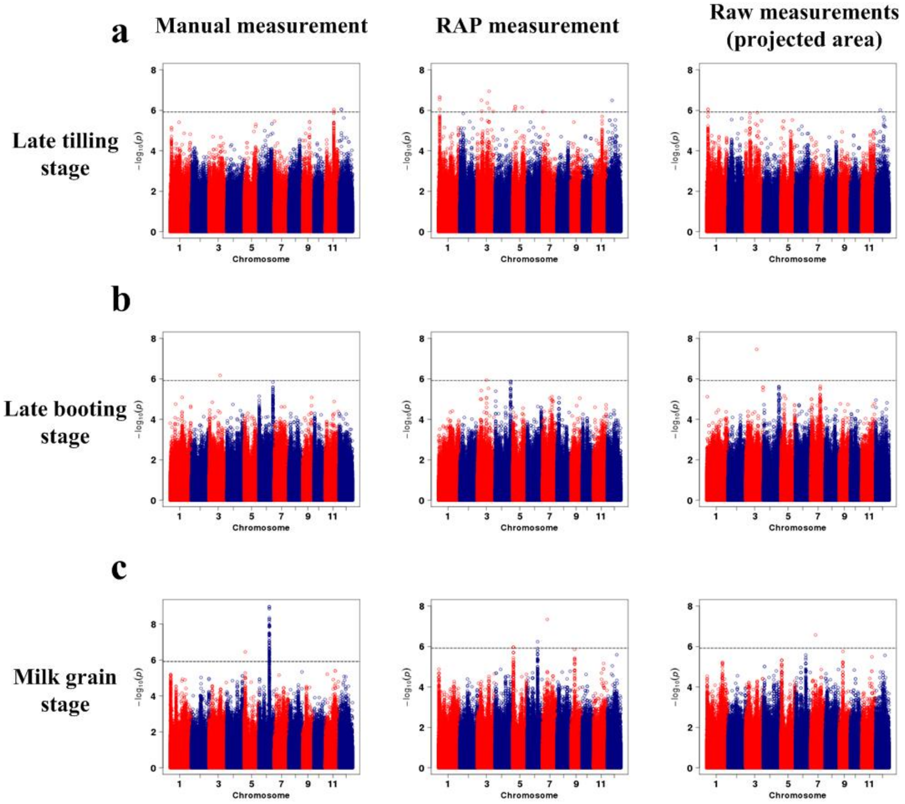

Supplementary Figure 5 Genome-wide association studies of shoot dry weight at three developmental stages using different phenotyping methods. Manhattan plots for shoot dry weight at the late tillering stage (a), late booting stage (b), and milk grain stage (c) using manual measurement (left), RAP measurement (middle), and raw measurement (right) (*P* = 1.21×10^{-6}). Sample sizes are 423 (a), 402 (b), and 269 (c), respectively. The *P* values are computed from a likelihood ratio test with a mixed-model approach using the factored spectrally transformed linear mixed models (FaST-LMM) program.

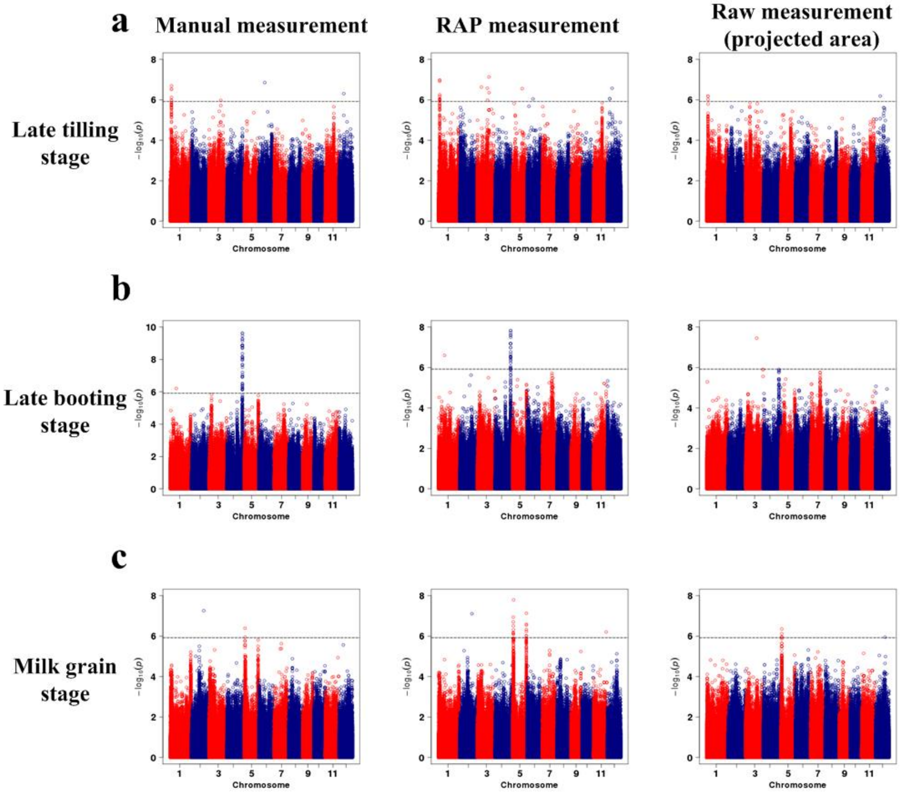

Supplementary Figure 6 Genome-wide association studies of green leaf area at three developmental stages using different phenotyping methods. Manhattan plots for shoot dry weight at the late tillering stage (a), late booting stage (b), and milk grain stage (c) using manual measurement (left), RAP measurement (middle), and raw measurement (right) (P = 1.21×10^{-6}). Sample sizes are 423 (a), 402 (b), and 269 (c), respectively. The P values are computed from a likelihood ratio test with a mixed-model approach using the factored spectrally transformed linear mixed models (FaST-LMM) program.

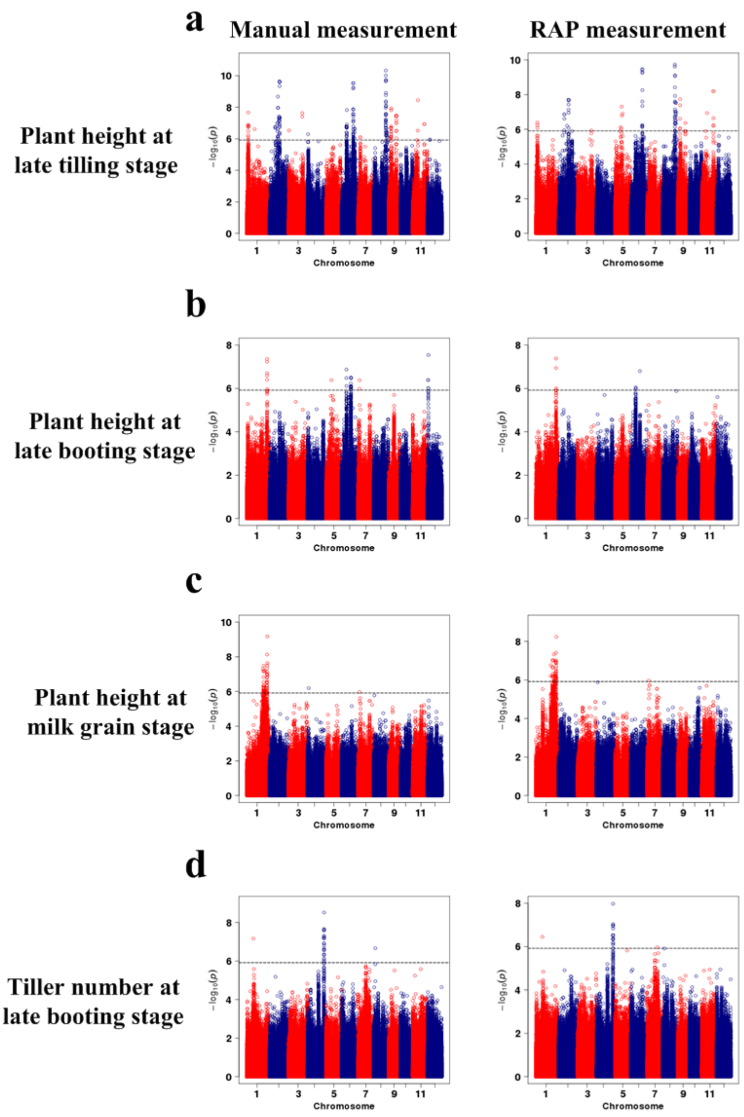

Supplementary Figure 7 Genome-wide association studies of plant height and tiller number using different phenotyping methods. Manhattan plots for plant height at the late tillering stage (a), plant height at the late booting stage (b), plant height at the milk grain stage (c), and tiller number at the late booting stage (d) using manual measurement (left) and RAP measurement (right) (P = 1.21×10^{-6}). Sample sizes are 423 (a), 402 (b), 269 (c), and 402 (d), respectively. The P values are computed from a likelihood ratio test with a

mixed-model approach using the factored spectrally transformed linear mixed models (FaST-LMM) program.

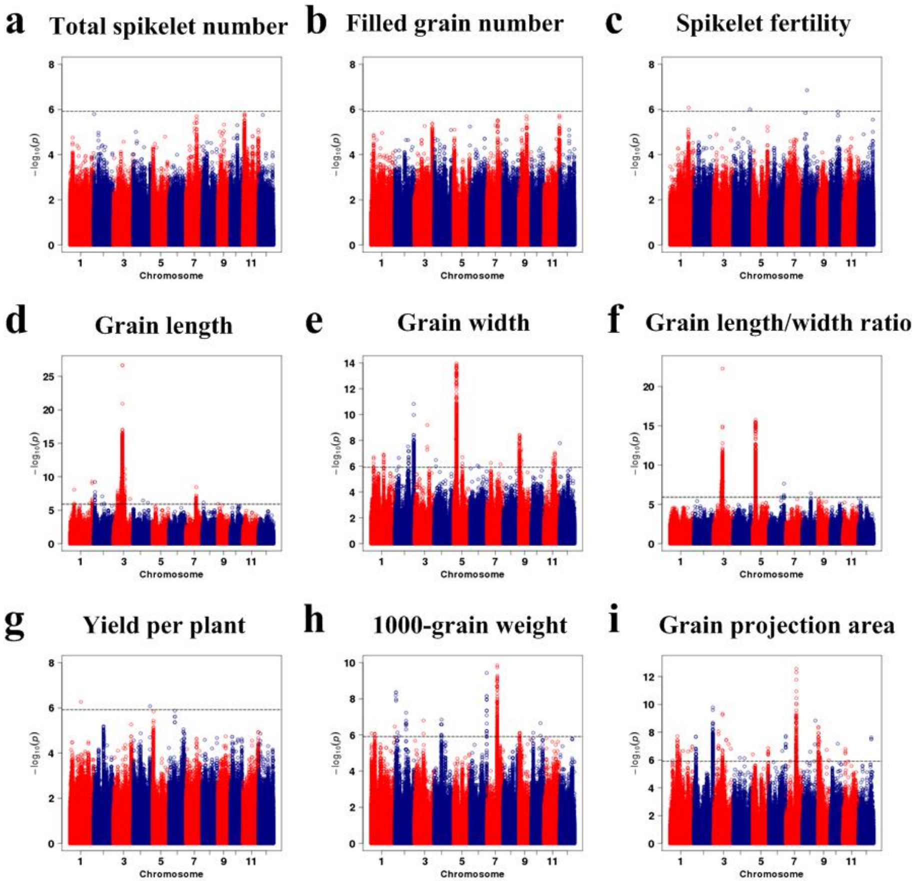

Supplementary Figure 8 Genome-wide association studies of yield-related traits measured by YTS. Manhattan plots for total spikelet number (a), filled grain number (b), spikelet fertility (c), grain length (d), grain width (e), grain length/width ratio (f), yield per plant (g), 1000-grain weight (h) and grain projected area (i) (P = 1.21×10^{-6}). Sample sizes are all 514. The P values are computed from a likelihood ratio test with a mixed-model approach using the factored spectrally transformed linear mixed models (FaST-LMM) program.

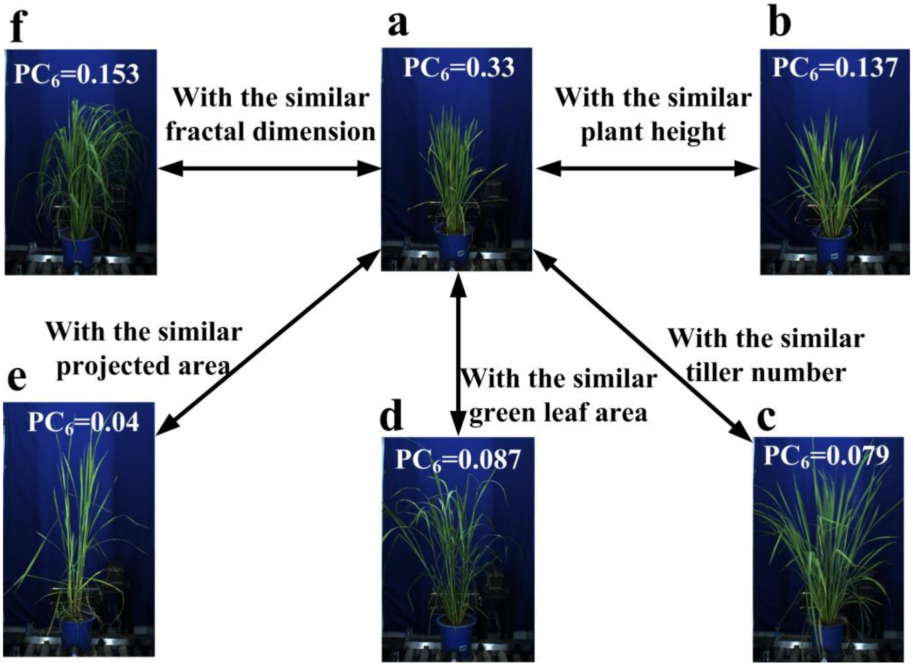

Supplementary Figure 9 Comparison of plant compactness with other similar phenotypic traits at the late booting stage. Comparison of plant compactness in plants with a similar plant height (a and b), similar tiller number (a and c), similar green leaf area (a and d), similar projected area (a and e), and similar fractal dimension (a and f). With similar phenotypic traits (such as plant height, tiller number, green leaf area, and so on), the rice plant with a higher PC value is more compact and the leaf angle becomes smaller.

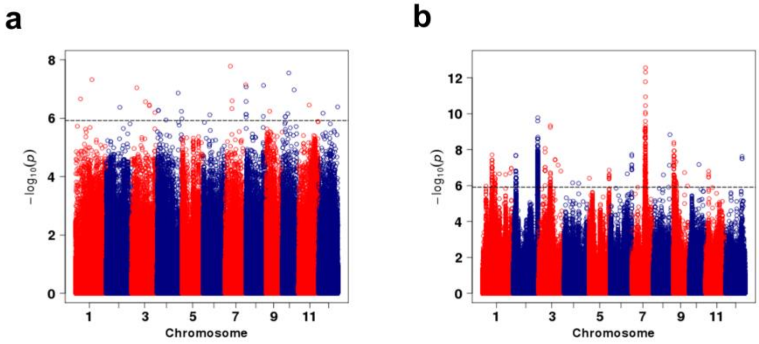

Supplementary Figure 10 GWAS results improvement of grain projected area after removing a phenotypic outlier. Manhattan plots for grain projected area with a phenotypic outlier (a) and without the outlier (b) ($P = 1.21\times10^{-6}$). The emergence of the outlier (from the accession C061) was due to the manual operation mistake with the YTS. The *P* values are computed from a likelihood ratio test with a mixed-model approach using the factored spectrally transformed linear mixed models (FaST-LMM) program.

Supplementary Table 1 Phenotyping accuracy of rice phenotypic traits with RAP and YTS*.

Traits	Stage	Selected model	Sample size	Training set			Testing set			5-fold cross validation		
				Adjusted R^2	MAPE	SD_{APE}	R^2	MAPE	SD_{APE}	R^2	MAPE	SD_{APE}
Green leaf area	LT	$Ln(GLA_M)=0.99lnA+2.40PC_6-0.14$	423	0.93	9.14%	10.21%	0.897	9.02%	8.49%	0.894	9.10%	9.71%
	LB	$Ln(GLA_M)=lnA+5.35F_{12}-0.08$	402	0.94	8.94%	8.11%	0.898	10.52%	8.69%	0.908	9.79%	8.58%
	MG	$Ln(GLA_M)=1.05lnA+1.99PC_6-0.03G\_g-0.31$	269	0.92	10.31%	10.56%	0.846	10.13%	9.35%	0.880	10.36%	10.22%
Shoot fresh weight	LT	$Ln(SFW_M)=1.01lnA-0.04G\_g-7.19$	423	0.91	10.24%	8.16%	0.89	10.78%	9.52%	0.89	10.43%	8.66%
	LB	$Ln(SFW_M)=0.99lnA+2.37F_{12}-7.46$	402	0.87	10.71%	9.34%	0.85	12.40%	11.85	0.87	11.67%	10.57%
	MG	$Ln(SFW_M)=1.03lnA+2.73F_{12}-7.63$	269	0.90	11.27%	8.15%	0.87	11.84%	8.12%	0.87	11.79%	8.30%
Shoot dry weight	LT	$Ln(SDW_M)=1.12lnA+2.18F_{12}-10.85$	423	0.91	10.13%	8.07%	0.89	10.75%	10.21%	0.88	10.40%	8.99%
	LB	$Ln(SDW_M)=0.78lnA+1.72PC_6+0.001MU3-6.77$	402	0.79	13.05%	10.55%	0.82	12.70%	10.14	0.79	12.92%	10.38%
	MG	$Ln(SDW_M)=1.04lnA+2.72F_{11}-8.89$	269	0.84	14.14%	11.84%	0.84	13.28%	10.10%	0.82	13.99%	11.07%
Plant height	LT, LB, MG		1094	0.94	5.59%	4.93%						
Tiller number	LB		402	0.95	8.88%	9.67 %						
Total spikelets			272	0.99	1.28%	1.24%						
Filled spikelets		-	272	0.99	0.89%	0.97%				-		
Grain length	After harvest		272	0.97	1.99%	1.53%						
Grain width			272	0.96	2.52%	2.09%						
1000-Grain weight			272	0.99	1.47%	0.98%						

* GLA_M, SFW_M, and SDW_M are manual measurements of plant height, green leaf area, shoot fresh weight, and shoot dry weight, respectively. During late tillering stage (LT), late booting stage (LB), and milk grain stage (MG), 423 rice samples, 402 rice samples, and 269 rice samples were inspected, respectively. More details

about the features extraction for modeling can be found in the Supplementary Table 2, the Supplementary Note 1, and the Supplementary Note 2. To choose the best model, all-possible regression was performed and more details will be shown in the Supplementary Fig. 3 and Supplementary Table 3-11. After harvest, to evaluate the measurement accuracy of the YTS, 68 rice accessions (with 4 replications) were randomly chosen and measured with manual methods and YTS, respectively.

Supplementary Table 2 The 33 features classification for modeling. More details about the features extraction can be found in the Supplementary Note 1 and the Supplementary Note 2.

Feature classification	Feature	Feature abbreviation	Feature definition
Projected area	Projected area	*A*	Seen in the Supplementary Note. 2
Morphological feature	Fractal dimension	*FD*	
	Average height of the bounding rectangle of the object	*H*	
	Average width of the bounding rectangle of the object	*W*	
	Tiller number	*TN*	
	Maximum natural plant height	*PH*	
	Maximum vertical plant height	*PH_V*	
	Maximum plant width	*PW*	
	Plant compactness	PC_1-PC_6	
	Relative frequencies	(F_1~F_{12})	
Texture feature	Mean gradient of the G image	*G_g*	
	the mean value	*M*	
	the standard error	*SE*	
	the third moment	*MU3*	
	the uniformity	*U*	
	the smoothness	*S*	
	the entropy	*E*	

Supplementary Table 3 Model selection of green leaf area at the late tillering stage. (the model with the blue color means the selected model with better performance and less predictors). All subsets regression and 5-flod cross validation were used for selecting the best model.

	Sample size	Variables in Model	Training set					Validation set			5-fold cross validation		
			Adjusted R^2	AIC	PRESS	MAPE	SD_{APE}	R^2	MAPE	SD_{APE}	R^2	MAPE	SD_{APE}
A	423	*A*	0.8394	-698.5123	7.67257	14.51%	13.51%	0.796	13.79%	11.17%	0.766	14.42%	12.81%
AT	423	*A, U*	0.8951	-787.2913	5.05487	11.87%	11.63%	0.843	11.57%	10.95%	0.843	11.79%	11.57%
AM	423	A, PC_6	0.9273	-864.8419	3.49817	9.14%	10.21%	0.897	9.02%	8.49%	0.894	9.10%	9.71%
ATM	423	A, PC_6, G_g	0.9312	-875.2764	3.34058	8.93%	10.03%	0.910	8.33%	8.16%	0.906	8.66%	9.46%

Supplementary Table 4 Model selection of green leaf area at the late booting stage. (the model with the blue color means the selected model with better performance and less predictors). All subsets regression and 5-flod cross validation were used for selecting the best model.

	Sample size	Variables in Model	Training set					Validation set			5-fold cross validation		
			Adjusted R^2	AIC	PRESS	MAPE	SD_{APE}	R^2	MAPE	SD_{APE}	R^2	MAPE	SD_{APE}
A	402	*A*	0.7801	-620.7954	9.13742	17.18%	11.98%	0.718	17.91%	14.22%	0.733	17.60%	13.00%
AT	402	*A, G_g*	0.8523	-699.8215	6.18215	13.82%	11.02%	0.807	14.18%	10.63%	0.817	14.07%	10.91%
AM	402	A, F_{12}	0.9363	-868.9169	2.67393	8.94%	8.11%	0.898	10.52%	8.69%	0.908	9.79%	8.58%
ATM	402	A, F_{12}, G_g	0.9405	-881.4876	2.51721	8.49%	8.05%	0.903	9.59%	8.22%	0.913	9.08%	8.32%

Supplementary Table 5 Model selection of green leaf area at the milk grain stage. (the model with the blue color means the selected model with better performance and less predictors). All subsets regression and 5-flod cross validation were used for selecting the best model.

	Sample size	Variables in Model	Training set					Validation set			5-fold cross validation		
			Adjusted R^2	AIC	PRESS	MAPE	SD_{APE}	R^2	MAPE	SD_{APE}	R^2	MAPE	SD_{APE}
A	269	*A*	0.7043	-359.9602	9.11255	21.29%	17.78%	0.499	20.01%	15.52%	0.559	21.05%	17.32%
AT	269	*A, G_g*	0.8501	-449.9721	4.66373	14.75%	12.60%	0.729	13.51%	11.50%	0.791	14.32%	12.41%
AM	269	*A, PC6*	0.8667	-465.7762	4.15286	12.78%	13.06%	0.795	12.39%	11.02%	0.822	12.76%	12.49%
ATM	269	*A*, PC_6, *G_g*	0.9167	-527.7066	2.63292	10.31%	10.56%	0.846	10.13%	9.35%	0.880	10.36%	10.22%

Supplementary Table 6 Model selection of shoot fresh weight at the late tillering stage. (the model with the blue color means the selected model with better performance and less predictors). All subsets regression and 5-flod cross validation were used for selecting the best model.

	Sample size	Variables in Model	Training set					Validation set			5-fold cross validation		
			Adjusted R^2	AIC	PRESS	MAPE	SD_{APE}	R^2	MAPE	SD_{APE}	R^2	MAPE	SD_{APE}
A	423	*A*	0.87	-780.59	5.25	12.60%	9.93%	0.87	12.07%	10.69%	0.84	12.25%	10.05%
AT	423	*A, G_g*	0.91	-861.95	3.58	10.24%	8.16%	0.89	10.78%	9.52%	0.89	10.43%	8.66%
AM	423	*A, TN*	0.90	-824.41	4.30	11.18%	9.12%	0.89	11.10%	11.09%	0.87	11.05%	9.62%
ATM	423	*A, TN, G_g*	0.92	-875.29	3.38	9.81%	7.95%	0.91	10.29%	9.68%	0.90	9.99%	8.54%

Supplementary Table 7 Model selection of shoot fresh weight at the late booting stage. (the model with the blue color means the selected model with better performance and less predictors). All subsets regression and 5-flod cross validation were used for selecting the best model.

	Sample size	Variables in Model	Training set					Validation set			5-fold cross validation		
			Adjusted R^2	AIC	PRESS	MAPE	SD_{APE}	R^2	MAPE	SD_{APE}	R^2	MAPE	SD_{APE}
A	402	*A*	0.83	-726.40	5.42	12.96%	10.25%	0.82	13.78%	13.80%	0.83	13.35%	11.88%
AT	402	*A*, *G_g*	0.84	-743.50	4.99	12.41%	9.75%	0.83	13.18%	12.89%	0.84	12.85%	11.37%
AM	402	*A*, F_{12}	0.87	-787.01	4.01	10.71%	9.34%	0.85	12.40%	11.85%	0.87	11.67%	10.57%
ATM	402	*A*, PC_6, *E*	0.88	-794.48	3.85	10.54%	8.75%	0.87	11.57%	11.76%	0.87	11.18%	10.29%

Supplementary Table 8 Model selection of shoot fresh weight at the milk grain stage. (the model with the blue color means the selected model with better performance and less predictors). All subsets regression and 5-flod cross validation were used for selecting the best model.

	Sample size	Variables in Model	Training set					Validation set			5-fold cross validation		
			Adjusted R^2	AIC	PRESS	MAPE	SD_{APE}	R^2	MAPE	SD_{APE}	R^2	MAPE	SD_{APE}
A	269	*A*	0.8671	-492.2718	3.40191	12.86%	9.10%	0.81	12.81%	9.30%	0.82	13.10%	9.35%
AT	269	*A*, *G_g*	0.8773	-501.9616	3.15330	12.61%	8.68%	0.83	12.12%	9.36%	0.84	12.62%	9.21%
AM	269	*A*, F_{12}	0.8952	-523.1669	2.71042	11.27%	8.15%	0.87	11.84%	8.12%	0.87	11.79%	8.30%
ATM	269	*A*, *G_g*, F_{12}	0.8975	-525.1457	2.66018	11.25%	8.07%	0.87	11.75%	8.19%	0.87	11.74%	8.32%

Supplementary Table 9 Model selection of shoot dry weight at the late tillering stage. (the model with the blue color means the selected model with better performance and less predictors). All subsets regression and 5-flod cross validation were used for selecting the best model.

	Sample size	Variables in Model	Training set					Validation set			5-fold cross validation		
			Adjusted R^2	AIC	PRESS	MAPE	SD_{APE}	R^2	MAPE	SD_{APE}	R^2	MAPE	SD_{APE}
A	423	*A*	0.90	-819.88	4.35	11.39%	8.63%	0.87	11.75%	10.59%	0.85	11.55%	9.54%
AT	423	*A*, *G_g*	0.91	-849.73	3.79	10.33%	8.17%	0.89	11.12%	10.05%	0.87	10.68%	9.10%
AM	423	*A*, F_{12}	0.91	-860.67	3.59	10.13%	8.07%	0.89	10.75%	10.21%	0.88	10.40%	8.99%
ATM	423	*A*, *G_g*, F_{12}	0.92	-869.41	3.45	9.69%	8.01%	0.90	10.53%	10.01%	0.89	10.07%	8.95%

Supplementary Table 10 Model selection of shoot dry weight at the late booting stage. (the model with the blue color means the selected model with better performance and less predictors). All subsets regression and 5-flod cross validation were used for selecting the best model.

	Sample size	Variables in Model	Training set					Validation set			5-fold cross validation		
			Adjusted R^2	AIC	PRESS	MAPE	SD_{APE}	R^2	MAPE	SD_{APE}	R^2	MAPE	SD_{APE}
A	402	*A*	0.67	-628.50	8.84	16.41%	14.38%	0.67	17.39%	15.74%	0.65	17.11%	14.67%
AT	402	*A, M*	0.72	-659.34	7.59	15.50%	12.81%	0.72	15.62%	13.49%	0.69	15.80%	13.09%
AM	402	*A*, PC_3	0.74	-675.39	7.00	14.27%	12.03%	0.76	13.90%	12.27%	0.73	14.32%	11.87%
ATM	402	*A*, PC_6, *MU3*	0.79	-718.35	5.66	13.05%	10.55%	0.82	12.70%	10.14%	0.79	12.92%	10.38%

Supplementary Table 11 Model selection of shoot dry weight at the milk grain stage. (the model with the blue color means the selected model with better performance and less predictors). All subsets regression and 5-flod cross validation were used for selecting the best model.

	Sample size	Variables in Model	Training set					Validation set			5-fold cross validation		
			Adjusted R^2	AIC	PRESS	MAPE	SD_{APE}	R^2	MAPE	SD_{APE}	R^2	MAPE	SD_{APE}
A	269	*A*	0.8229	-441.6034	4.97151	14.97%	12.21%	0.81	13.93%	10.24%	0.80	14.56%	11.34%
AT	269	*A, M*	0.8331	-448.5874	4.71907	14.63%	11.88%	0.82	13.26%	10.19%	0.81	14.13%	11.23%
AM	269	A, F_{11}	0.8388	-453.24	4.55337	14.14%	11.84%	0.84	13.28%	10.10%	0.82	13.99%	11.07%
ATM	269	$A, F_{11}, MU3$	0.8530	-464.6470	4.18673	13.90%	10.47%	0.84	12.93%	9.71%	0.83	13.64%	10.45%

Supplementary Table 12 Main specifications of RAP inspection unit.

Inspection device	characteristic	
Color imaging device	Manufacturer of the color camera	Stingray F-504C, Allied Vision Technologies, Germany
	Resolution of the color camera	2452 (height) × 2056 (width)
	Focal length of the lens	8 mm
	Field of view	1824 mm (height) × 1529 mm (width)
	Acquired image number per plant	12 images
	Image size per plant	67.2 Megabytes
Linear X-ray CT	Manufacturer of the X-ray source	T80-1-60, BMEI Co. Ltd, China
	Manufacturer of the X-ray detector	X-scan 0.4f3-205, X-Scan Imaging Corporation, USA
	X-ray tube voltage	50 KV
	X-ray tube current	1 mA
	Resolution of the section image	0.32 mm × 0.32 mm
	Transaxial field of view	307 mm
	Acquired image number per plant	1 sinogram image
	Image size per plant	500 Kilobytes
	More details were listed in the Supplementary Information References 13.	

Supplementary Table 13 Information about all potential candidate genes within 200 kb (100kb upstream and downstream) of the lead SNP 0431688161 detected by RAP.

Start	End	Description	LOC
31592111	31595891	expressed protein;	LOC_Os04g53360
31601482	31603139	acyltransferase__putative__expressed;	LOC_Os04g53370
31603302	31615895	expressed protein;	LOC_Os04g53380
31616827	31618125	MrBTB2 - Bric-a-Brac__Tramtrack__Broad Complex BTB domain with Meprin and TRAF Homology MATH-related domain;	LOC_Os04g53390
31624601	31625728	MBTB6 - Bric-a-Brac__Tramtrack__Broad Complex BTB domain with Meprin and TRAF Homology MATH domain;	LOC_Os04g53400
31626911	31628011	MBTB7 - Bric-a-Brac__Tramtrack__Broad Complex BTB domain with Meprin and TRAF Homology MATH domain__expressed;	LOC_Os04g53410
31629865	31630320	hypothetical protein;	LOC_Os04g53420
31632518	31633639	MBTB8 - Bric-a-Brac__Tramtrack__Broad Complex BTB domain with Meprin and TRAF Homology MATH domain__expressed;	LOC_Os04g53430
31634353	31638374	RNA recognition motif containing protein__putative__expressed;	LOC_Os04g53440
31638980	31643819	expressed protein;	LOC_Os04g53450
31646000	31651374	AT hook motif family protein__expressed;	LOC_Os04g53460
31653036	31656964	expressed protein;	LOC_Os04g53470
31658054	31661806	transposon protein__putative__unclassified;	LOC_Os04g53480
31663387	31665762	OsCHL Chloroplastic lipocalin__expressed;	LOC_Os04g53490
31669323	31677180	NBS-LRR disease resistance protein__putative;	LOC_Os04g53496
31679078	31682304	expressed protein;	LOC_Os04g53502
31690620	31694644	OsFBL20 - F-box domain and LRR containing protein__expressed;	LOC_Os04g53510
31695388	31709470	expressed protein;	LOC_Os04g53520
31714613	31722017	homeobox and START domains containing protein__putative__expressed;	LOC_Os04g53540
31710147	31715587	expressed protein;	LOC_Os04g53530
31725145	31725462	transposon protein__putative__unclassified;	LOC_Os04g53544
31740550	31743491	ABC transporter__ATP-binding protein__putative;	LOC_Os04g53550
31747495	31748604	expressed protein;	LOC_Os04g53560
31752100	31754088	P21-Rho-binding domain containing protein__putative__expressed;	LOC_Os04g53580

31760868	31766827	retrotransposon protein__putative__Ty3-gypsy subclass;	LOC_Os04g53590
31771101	31772442	hypothetical protein;	LOC_Os04g53600
31772548	31773525	expressed protein;	LOC_Os04g53606
31774421	31776771	APO__putative__expressed;	LOC_Os04g53612
31777433	31780201	ubiquitin family protein__putative__expressed;	LOC_Os04g53620
31781356	31783194	pentatricopeptide__putative__expressed;	LOC_Os04g53630
31785735	31787713	peroxidase precursor__putative__expressed;	LOC_Os04g53640

Supplementary Table 14 Information about all potential candidate genes within 200 kb (100kb upstream and downstream) of the lead SNP 0718815103 detected by YTS.

Start	End	Description	LOC
18716041	18724775	ubiquitin family protein__putative__expressed;	LOC_Os07g31540
18726995	18732575	powdery mildew resistant protein 5__putative__expressed;	LOC_Os07g31550
18739392	18740416	hypothetical protein;	LOC_Os07g31560
18742405	18742851	hypothetical protein;	LOC_Os07g31570
18754264	18758141	retrotransposon protein__putative__Ty1-copia subclass;	LOC_Os07g31590
18760294	18762732	expressed protein;	LOC_Os07g31599
18764680	18766147	peroxidase precursor__putative__expressed;	LOC_Os07g31610
18771517	18773556	transposon protein__putative__Pong sub-class;	LOC_Os07g31620
18778332	18783482	retrotransposon protein__putative__unclassified;	LOC_Os07g31630
18784106	18787328	retrotransposon protein__putative__unclassified;	LOC_Os07g31640
18799975	18805196	expressed protein;	LOC_Os07g31650
18805655	18806800	conserved hypothetical protein;	LOC_Os07g31660
18809001	18810077	hypothetical protein;	LOC_Os07g31670
18811316	18812764	OsFBL38 - F-box domain and LRR containing protein__expressed;	LOC_Os07g31680
18813667	18817142	retrotransposon protein__putative__unclassified__expressed;	LOC_Os07g31690
18820316	18829346	transposon protein__putative__CACTA__En/Spm sub-class;	LOC_Os07g31700
18829963	18831098	hypothetical protein;	LOC_Os07g31710
18839254	18841544	GTPase activating protein__putative__expressed;	LOC_Os07g31720
18846298	18846612	hypothetical protein;	LOC_Os07g31740
18851479	18855439	chalcone synthase__putative;	LOC_Os07g31750
18866712	18869400	hypothetical protein;	LOC_Os07g31760
18872797	18874933	chalcone synthase__putative__expressed;	LOC_Os07g31770
18878388	18884107	retrotransposon protein__putative__unclassified;	LOC_Os07g31780
18892449	18893795	CXE carboxylesterase__putative;	LOC_Os07g31790
18894761	18903542	NB-ARC domain containing protein__expressed;	LOC_Os07g31800
18905415	18905777	conserved hypothetical protein;	LOC_Os07g31810
18910794	18913898	GTPase activating protein__putative__expressed;	LOC_Os07g31830

18913951	18919567	leucine-rich repeat family protein__putative__expressed;	LOC_Os07g31840

Supplementary Table 15 Comparison of GWAS results using different measurement methods for shoot fresh/dry weight and green leaf area.

Traits	Stage	Chr.	Mannual measurement		RAP measurement		Raw measurement	
			Position(MSU6.1)[a]	*P* value	Position(MSU6.1)[a]	*P* value	Position(MSU6.1)[a]	*P* value
shoot fresh weight	late tillering stage	11	**17693188**	9.39E-08	**17693188**	9.14E-07		
		1			2578017	2.46E-07		
	late booting stage	4	**31688161**	1.45E-07	**31694687**	1.35E-07		
			31452496	5.86E-07				
	milk grain stage	5	1982152	1.60E-07	2153885	6.63E-07		
			1796873	3.47E-07				
		6	**21523087**	1.98E-09	**21523087**	2.55E-07		
shoot dry weight	late tillering stage	11	17699747	9.21E-07				
		1			2578017	2.25E-07		
	milk grain stage	6	**21505123**	1.07E-09	**21523087**	5.77E-07		
		5			2153885	1.09E-06		
green leaf area	late tillering stage	1	**2578034**	2.02E-07	**2578017**	1.06E-07	**2578017**	6.62E-07
	late booting stage	4	**31694687**	2.41E-10	**31694687**	1.50E-08		
					31305224	1.01E-06		
	milk grain stage	5	**1852260**	4.07E-07	**1852260**	1.15E-07	2231657	4.43E-07
			**1982152**	1.13E-06	**1982152**	3.07E-07		
					2142701	1.63E-08		
					28506264	7.37E-08		

[a] Numbers about position of lead SNPs in bold in the same row indicated that corresponding SNPs were the same SNPs or in high LD (r^2 >0.8 except r^2 (22287733 & 22287972)=0.57).

Supplementary Table 16 Effective number of SNPs across rice genome and LD-adjusted Bonferroni corrected *P* value thresholds.

**Chromosome**	**Observed_Number**	**Effective_Number**	**Effective_Ratio**	**Suggestive_*P*_Value**	**Significant_*P* _Value**
1	498272	87561	0.18	1.14E-05	5.71E-07
2	387222	67305	0.17	1.49E-05	7.43E-07
3	381693	60705	0.16	1.65E-05	8.24E-07
4	372273	76170	0.20	1.31E-05	6.56E-07
5	309780	50767	0.16	1.97E-05	9.85E-07
6	364052	65485	0.18	1.53E-05	7.64E-07
7	343840	64962	0.19	1.54E-05	7.70E-07
8	345123	70740	0.20	1.41E-05	7.07E-07
9	278739	55701	0.20	1.80E-05	8.98E-07
10	316925	61777	0.19	1.62E-05	8.09E-07
11	398281	89597	0.22	1.12E-05	5.58E-07
12	362400	78681	0.22	1.27E-05	6.35E-07
Genome	4358600	829451	0.19	1.21E-06	6.03E-08

Supplementary Table 17 Imaging techniques used in RAP and YTS.

	Phenotyping elements	Phenotypic traits	Imaging approaches
High-throughput rice phenotyping facility (HRPF)	Rice automatic phenotyping (RAP)	Plant height	Color imaging device
		Green leaf area	Color imaging device
		Shoot fresh weight	Color imaging device
		Shoot dry weight	Color imaging device
		Plant compactness	Color imaging device
		Tiller number	Linear X-ray CT
	Yield traits scorer (YTS)	Total spikelet number	Line-scan imaging
		Filled grain number	Line-scan imaging
		Spikelet fertility	Line-scan imaging
		Yield per plant	Electronic balance
		Grain length	Line-scan imaging
		Grain width	Line-scan imaging
		Grain length/width ratio	Line-scan imaging
		1000-grain weight	Line-scan imaging and electronic balance
		Grain projected area	Line-scan imaging

Supplementary Note 1. Color component extraction

To enhance the plant region extraction, the image was transformed to an excessive green (ExG) image and an excessive red (ExR) image using:

$$ExG=2Ng-Nr-Nb \tag{1}$$

$$ExR=1.4Nr-Nb \tag{2}$$

where Ng, Nr, Nb was the normalized r, g, b component, defined by the Equation 3-5.

$$Nr=\frac{R}{R+G+B} \tag{3}$$

$$Ng=\frac{G}{R+G+B} \tag{4}$$

$$Nb=\frac{B}{R+G+B} \tag{5}$$

where R, G and B are the grayscale values for each RGB channel.

And i2 component was extracted using the following equation.

$$i2=\frac{R-B}{2} \tag{6}$$

where R and B are the grayscale values for R, B channel.

Supplementary Note 2. Definition of the features

- Projected area (*A*): Number of foreground pixels.
- Fractal dimension (*FD*): Superimpose boxes with box size of δ_k on the interested object, and calculate the number of boxes that are needed to cover the object, denoted as N_{δ_k}. Repeat this process with reducing δ_k until δ_k approaches pixel size. Fractal dimension was calculated using the following Equation.

$$FD=\lim_{\delta_k \to 0}\frac{\ln N_{\delta_k}}{-\ln \delta_k} \tag{7}$$

- Height of the bounding rectangle (*H*) of the object.
- Width of the bounding rectangle (*W*) of the object.
- Tiller number (*TN*) :Extracted by the X-ray CT system (seen in Supplementary Information Reference 12).
- Natural plant height (*PH*): Plant height under natural condition, the maximum length from the top leaf blade to the soil surface when the rice plant is straightened.
- Vertical plant height (*PH_V*): The height of the bounding rectangle of the rice plant.
- Plant width (*PW*): The width of the bounding rectangle of the rice plant.
- Plant compactness (PC_1-PC_6)[14]: Divide the image into several sub-images using a (5 × 5) window. And calculate the ratio of the foreground pixels to the total number of pixels in each sub-image (5 × 5), denoted as plant compactness in each sub-image (*PCs*). Categorize *PCs* into six classes: C1: <10%, C2: 10-20%, C3: 20-40%, C4: 40-60%, C5: 60-80%, C6: 80-100%. Then Count the number of *PCs* belonging to each class, denoted as ND_i

(i=1,2…6). At last, leaf compactness of class i (PC_i) was computed as the percentage of ND_i compared to the sum of ND_i.

- Relative frequencies(F_1~F_{12}): calculated as the following steps:

$$I=\sum_{i=1}^{12} BW_i \tag{8}$$

Where BWi is the binary image of the ith side-view image. I is the grayscale image of the sum of the 12 binary images.

$$F(i) = number(I_{(x,y)} = i), i = 1,2...12 \tag{9}$$

Then the pixels was then categorized into 12 classes. Calculate number of pixels in each class, denoted as $F(i)$ (i=1,2…12) . Relative frequencies(F_1~F_{12}) was computed using the following equation.

$$F_i = \frac{F_{(i)}}{\sum F_{(i)}} \quad (i=1,2...12) \tag{10}$$

- Mean gradient of the G image (G_g)
- The six histogram features, including the mean value (M), the standard error (SE), the third moment ($MU3$), the uniformity (U), the smoothness(S) and the entropy (E), were calculated using the following equations.

$$M = \sum_{i=0}^{L-1} G_i p(G_i) \tag{11}$$

$$SE = \sqrt{\sum_{i=0}^{L-1} (G_i - M)^2 p(G_i)} \tag{12}$$

$$MU3 = \sum_{i=0}^{L-1} (G_i - M)^3 p(G_i) \tag{13}$$

$$U = \sum_{i=0}^{L-1} p^2(G_i) \tag{14}$$

$$S = 1 - \frac{1}{1 + SE^2} \tag{15}$$

$$E = \sum_{i=0}^{L-1} p(G_i)\log_2 p(G_i) \tag{16}$$

Where G_i was the i-th graylevel, and $p(G_i)$ was the probability of G_i. L was the maximum gray level.

- Grain projected area (GPA): First the acquired gray-scale images were segmented to extract the rice grains from the background. Then the pixel number of each grain in the binary image was counted and regarded as grain projected area. Finally the GPA of the rice accession was defined by the average value of the projected area of each rice grain.

Supplementary Note 3. Lead SNPs linked to known genes

For plant height, the lead SNP 0138417696/4.4E-08 (SNP ID/*P* value) was approximately 33 kb away from *SD1*[1-3],

which encodes the biosynthesis enzyme GA20ox to control rice plant height; SNP 0132203058/3.46 E-07 (in LD with lead SNP 0132002888/2.35E-07, r^2= 0.464) was approximately 17 kb away from *OsGH3-2*[4], a gene encoding an enzyme catalyzing IAA conjunction to amino acids and affecting plant height. Lead SNP 0609386264/1.09E-06 was approximately 49 kb away from *Hd1*[5], affecting heading date, plant height, and yield traits.

For plant compactness, lead SNP 0609320641/1.1E-06 was approximately 15 kb away from *Hd1*[5].

For grain-related traits, lead SNP 0234759816/1.49E-11, associated with grain width, and SNP 0234663116/1.20 E-08 (in high LD with the lead SNP 0234844459/1.65E-10, r^2= 0.770), associated with grain projected area, were approximately 76 kb and 19 kb away, respectively, from *TH1*[6], a gene controlling the grain shape and development of lemma and palea. Lead SNP 0316705162/2.28 E-27, associated with grain length; lead SNP 0316732087/5.27 E-23, associated with the grain length/width ratio; lead SNP 0316676687/1.58 E-07, associated with 1000-grain weight; and lead SNP 0316705162/4.60 E-10, associated with grain projected area, were approximately 23 kb, 0 kb, 51 kb, and 23 kb away, respectively, from *GS3*[7,8], a gene known to regulate grain size. Lead SNP 0505362462/9.75 E-08, associated with grain width, and lead SNP 0505360088/2.21 E-13, associated with the grain length/width ratio, were approximately 2 kb and < 1 kb away, respectively, from *qSW5*[9], a gene previously identified as controlling grain width. Lead SNP 0203807608/2.06 E-08, associated with grain projected area, was approximately 25 kb away from *MADS29*[10], a key regulator of early rice seed development.

For spikelet fertility, lead SNP 1226227332/2.83 E-06 was approximately 46 kb away from *OsPPKL3*, which is a homolog of *OsPPKL1*[11] and exhibits similar expression profiles and levels and has similar functions to *OsPPKL1*. *OsPPKL1* affects grain length, filling, and weight.

For yield-related traits, lead SNP 0332707137/4.51 E-06, associated with filled spikelet number, and lead SNP 0332717490/5.47 E-06, associated with yield per plant, were approximately 67 kb and 78 kb away, respectively, from *DST*[12], a gene previously identified as regulating grain number per panicle and grain yield.

Supplementary Note 4. New association signals detected by RAP and YTS

Strong association signals that had not been previously confirmed until now were detected in our study. Two newly associated loci are described as an example. A strong association signal on chromosome 4, underlain by lead SNP 0431688161 or lead SNP 0431694687 (the two SNPs were in high LD with r^2 = 0.879) was associated with shoot fresh weight, tiller number, and green leaf area at the late booting stage measured by RAP and manual measurements. Another strong association signal on chromosome 7, underlain by lead SNP 0718815103 or 0718804096 (the two SNPs were in approximately complete LD with r^2 = 0.967), was associated with grain length and 1000-grain weight measured by YTS. All potential candidate genes within 200 kb (100 kb upstream and downstream) of the lead SNP 0431688161 and lead SNP 0718815103 are listed in Supplementary Tables 14 and 15. Follow-up analysis based on gene annotation and expression data was required to investigate putative causal genes, and multiple methods, including resequencing, genetic transformation, mutant assay, and genetic linkage mapping, could be adopted to identify and validate causal variants underlying these loci.

Supplementary Note 5. Source of genotype information of the 533 accessions in our study

The genotype information of the 533 accessions was retrieved from the website "RiceVarMap" (http://ricevarmap.ncpgr.cn/).

Supplementary References

1. Sasaki, A. *et al.* A mutant gibberellin-synthesis gene in rice. *Nature* **416,** 701-702 (2002).
2. Monna, L. *et al.* Positional cloning of rice semidwarfing gene, *sd-1*: rice "green revolution gene" encodes a mutant enzyme involved in gibberellin synthesis. *DNA Res.* **9**, 11-17 (2002).
3. Spielmeyer, W., Ellis, M. H. & Chandler, P.M. Semidwarf (*sd-1*), ‘‘green revolution’’ rice, contains a defective gibberellin 20-oxidase gene. *Proc. Natl. Acad. Sci. U S A* **99**, 9043-9048 (2002).
4. Du, H. *et al*. A GH3 family member, OsGH3-2, modulates auxin and abscisic acid levels and differentially affects drought and cold tolerance in rice. *J. Exp. Bot.* **63,** 6467-6480 (2012).
5. Zhang, Z *et al*. Pleiotropism of the photoperiod-insensitive allele of Hd1 on heading date, plant height and yield traits in rice. *PLoS One* **7**, e52538 (2012).
6. Li, X. *et al*. *TH1*, a DUF640 domain-like gene controls lemma and palea development in rice. *Plant Mol. Biol.* **78**, 351-359 (2012).
7. Fan, C. *et al. GS3*, a major QTL for grain length and weight and minor QTL for grain width and thickness in rice, encodes a putative transmembrane protein. *Theor. Appl. Genet.* **112**, 1164-1171 (2006).
8. Mao, H. *et al*. Linking differential domain functions of the GS3 protein to natural variation of grain size in rice. *Proc. Natl. Acad. Sci. U S A* **107**, 19579–19584 (2010).
9. Shomura, A. *et al*. Deletion in a gene associated with grain size increased yields during rice domestication. *Nat. Genet.* **40**, 1023-1028 (2008).
10. Yin, L. & Xue, H. The MADS29 transcription factor regulates the degradation of the nucellus and the nucellar projection during rice seed development. *Plant Cell* **24**, 1049-1065 (2012).
11. Zhang, X. *et al*. Rare allele of OsPPKL1 associated with grain length causes extra-large grain and a significant yield increase in rice. *Proc. Natl. Acad. Sci. U S A* **109**, 21534–21539 (2012).
12. Li, S. *et al*. Rice zinc finger protein DST enhances grain production through controlling Gn1a/OsCKX2 expression. *Proc. Natl. Acad. Sci. U S A* **110**, 3167–3172 (2013).
13. Yang, W. *et al*. High-throughput measurement of rice tillers using a conveyor equipped with X-ray computed tomography. *Rev. Sci. Instrum.* **82**, 025102-025109 (2011).
14. Oka, M. and Hinata K. An application of computer image analysis for characterization of plant type in rice cultivars. *Japan J. Breed* **38**, 449-458 (1988).
15. Bradbury, P. J. *et al*. TASSEL: software for association mapping of complex traits in diverse samples. *Bioinformatics* **23,** 2633-2635 (2007).
